# The application of IS6110-baced loop-mediated isothermal amplification (LAMP) in the early diagnosis of tuberculous meningitis

**DOI:** 10.18632/oncotarget.15734

**Published:** 2017-02-25

**Authors:** Wen-Wen Sun, Qin Sun, Li-Ping Yan, Qing Zhang

**Affiliations:** ^1^ Clinic and Research Center of Tuberculosis, Shanghai Key Laboratory of Tuberculosis, Shanghai Pulmonary Hospital, Tongji University School of Medicine, Shanghai, China

**Keywords:** tuberculous meningitis (TBM), diagnosis, loop-mediated isothermal amplification

## Abstract

Here, we evaluated the potential activity of rapid *Mycobacterium tuberculosis* detection with loop-mediated isothermal amplification (LAMP), for the early diagnosis of tuberculous meningitis (TBM). Patients with suspected TBM from January 2014 to December 2015 were reviewed retrospectively. The cerebrospinalfluid(CSF) was collected. Acid-fast bacillus (AFB) staining, MGIT 960 culture, real-time fluorescent quantitative polymerase chain reaction (RTFQ PCR) and LAMP were performed. A total of 200 patients were included in the study. Of which, 172 of them were diagnosed with TBM (86.00%). The sensitivities of AFB staining, MGIT 960 culture, LAMP and RTFQ PCR for TBM diagnosis were 2.91% (5/172), 12.79% (22/172), 43.02% (74/172), and 34.30% (59/172), respectively. The sensitivity of LAMP for TBM was significantly higher than those of AFB staining and MGIT960 culture (*χ2* = 75.11, *P* < 0.001; *χ2* = 43.88, *P* = 0.002). LAMP's sensitivity was however comparable to RTFQ PCR assay (*χ2* = 2.08, *P* = 0.130). The specificity, positive predictive value and negative predictive value of LAMP in the diagnosis of TBM were 92.86% (26/28), 97.37% (74/76) and 20.97 % (26/124), respectively. The overall consistency between LAMP and RTFQ PCR in the diagnosis of TBM was 88.5% (177/200), with Kappa value of 0.870. The consistency between LAMP and MGIT960 culture was 71% (142/200), with Kappa value of 0.730. Among all the methods, LAMP had high sensitivity, specificity and positive predictive value, showing high consistency with MGIT960 culture and RTFQ PCR.

## INTRODUCTION

*Tuberculous meningitis* (TBM) patients often show no specific clinical manifestations. The positive rate of pathogen detection is low [1]. In addition, the change in cerebrospinal fluid (CSF) parameters is atypical, which is similar to those of new cryptococcal meningitis and viral meningitis [1]. Thus, early diagnosis of TBM is difficult [1]. Loop-mediated isothermal amplification (LAMP) is a new nucleic acid amplification method in the detection of *Mycobacterium tuberculosis* complex (MTBC) [2]. It is simple and sensitive method, which doesn't require expensive instruments and laboratory environments [3]. Existing studies have demonstrated its high diagnostic potential for detecting pulmonary and extrapulmonary Tuberculosis [3]. Few studies, however, have evaluated the activity of LAMP in the detection of *mycobacterium tuberculosis (MTB)* in CSF [2]. The study by Nagdev *et al*., has suggested that LAMP is a promising and accurate technique for the rapid diagnosis of TBM [2].

China has one of the highest *tuberculosis* (TB) burden in the world [4]. In the current study, a total of 200 patients with suspected TBM were enrolled. These patients received anti-TB therapy or diagnostic anti-TB therapy between January 2014 and December 2015, at Shanghai Pulmonary Hospital (Shanghai, China). The CSF was collected for the detection of *Mycobacterium tuberculosis* by acid-fast bacillus (AFB) staining, BACTEC™ MGIT960 culture (MGIT 960). Real-time fluorescent quantitative polymerase chain reaction (RTFQ PCR) analysis of LAMP was performed. The clinical value of LAMP for the diagnosis of TBM was assessed.

## RESULTS

### TBM diagnosis

TBM was diagnosed according to the diagnostic criteria developed by the TBM cooperation group in 2010 [5]. Of all 200 participants, 172 were diagnosed with TBM. There were 108 males and 64 females, with the mean age of 38.3 ± 22.8 year old (range: 16-78 year old). Twenty-eight patients were TBM negative (Viral meningitis: *n* = 16; Bacterial meningitis: *n* = 6; Cryptococcal meningitis: *n* = 4; Brain Metastases of Lung Cancer: *n* = 2). Of the 28 patients, 21 were males and 9 were females, with the mean age of 42.8 ± 24.5 year old (range: 21-76 year old).

In the 172 TBM patients, AFB, MGIT960 culture, LAMP and RTFQ PCR were performed for the diagnosis of TBM. The sensitivities of AFB, MGIT960 culture, LAMP and RTFQ PCR were 2.91% (5/172), 12.79% (22/172), 43.02% (74/172) and 34.30% (59/172), respectively. Significantly, the LAMP method had the shortest median time to final diagnosis (1 hour, Table 1).

**Table 1:** Sensitivity, specificity, positive predictive value and negative predictive value of 4 methods in the diagnosis of TBM

Group	TBM group (*n* = 172)	non-TBM group (*n* = 28)	Sensitivity (%)	specificity (%)	positive predictive value (%)	negative predictive value (%)	median time to final diagnosis
LAMP positive	74	2	43.02	92.86	97.37	20.97	1h
LAMP negative	98	26	——	——	——	——	——
RTFQ PCR positive	59	0	34.30	100	100	19.86	4h
RTFQ PCR negative	113	28	——	——	——	——	——
Acid-fast staining positive	5	0	2.91%	100	100	14.36	1d
Acid-fast staining negative	167	28	——	——	——	——	——
MGIT960 culture positive	22	0	12.79	100	100	15.73	41d
MGIT960 culture negative	150	28	——	——	——	——	——

**Table 2:** Comparison of LAMP with other methods

Group	LAMP positive (*n*)	LAMP negative (*n*)	Consistency (%)	Kappa value
RTFQPCR positive (*n*)	56	3	88.5	0.87
RTFQPCR negative (*n*)	20	121		
MGIT96 culture positive (*n*)	20	2	71	0.73
MGIT960 culture negative (*n*)	56	122		
Acid-fast staining positive (*n*)	4	1	63.5	0.59
Acid-fast staining negative (*n*)	72	123		

As compared with MGIT 960 culture method and acid-fast staining method, the sensitivity of LAMP in the diagnosis of *Mycobacterium tuberculosis* was significantly higher (*χ2* = 75.11, *P* = 0.000; *χ2* = 43.88, *P* = 0.002). Yet, LAMP's sensitivity was similar to that of RTFQ PCR (*χ2* = 2.08, *P* = 0.130).

### Comparison of LAMP with other methods

RTFQ PCR was employed as the gold standard in the diagnosis of *Mycobacterium tuberculosis*. In RTFQ PCR positive patients, the sensitivity of LAMP was 94.92% (56/59). Further, in PCR negative patients, the specificity of LAMP was 85.82 % (121/141). A decent consistency was observed between RTFQ PCR and LAMP (88.5%; 177/200; Kappa = 0.87). In MGIT 960 culture positive patients, the sensitivity of LAMP was 90.91% (20/22); in MGIT 960 culture negative patients, the specificity of LAMP was 68.54% (122/178). The consistency was 71% (142/200), and the Kappa value was 0.73. In acid-fast staining positive patients, the sensitivity of LAMP was 80% (4/5); In acid-fast staining negative patients, the specificity of LAMP was 65.08% (123/195). The consistency was 63.5% (127/200) with Kappa value of 0.59.

## DISCUSSION

TBM is an infectious disease of the central nervous system. It is characterized by the cerebrovascular and brain parenchyma lesions caused by the blood-borne spread of *Mycobacterium tuberculosis*, leading to subsequent implantation in the meninges, pia mater and the subarachnoid space. In many TBM patients, the clinical manifestations are atypical. Thus, to improve the early diagnosis of TBM is imperative. According to recent international diagnostic criteria for TBM [5], the presence of CSF AFB staining and RTFQ PCR are gold standards in clinical definite diagnosis of TBM [5]. However, the amount of bacilli in CSF is often very low, and acid-fast staining often displays low sensitivity and low specificity [5]. In addition, the dead bacilli is difficult to differentiate from the living bacilli [5]. Although bacilli culture is precise and reliable, it is time consuming, which could cause the loss of treatment time [6]. Current TBM clinical diagnosis relies on the clinical manifestations, CSF examination, imaging examinations and response to tentative anti-TB therapy [7]. In recent years, the non-standard applications of antibiotics and steroids may temporarily relieve TBM in some patients [10], which should also interfere the diagnosis of TBM. Thus, this study is performed to compare the methods of pathogen detection in the early diagnosis of TBM.

MGIT 960 culture is recently developed specifically for the rapid culture of *Mycobacterium tuberculosis* [8]. In MGIT 960 culture, the fluorescence intensity of the medium is continuously detected to evaluate the *Mycobacterium tuberculosis* growth. The mean detection time is significantly shorten after MGIT 960 culture (2-4 weeks). Therefore, MGIT 960 culture has been one of the most effective methods in the pathogen detection of TB [7, 9]. In the present study, MGIT 960 culture had the sensitivity of only 12.79% (22/172) in the diagnosis of TBM, which was yet still higher than that of acid-fast staining (2.91%, 5/172). The low sensitivity of MGIT 960 culture may not meet the clinical requirement for TBM diagnosis.

RTFQ PCR employs additional fluorescent probes in PCR, and integrates nucleic acid amplification, hybridization and spectral analysis [10–12]. This should significantly increase the sensitivity and specificity of pathogen detection. There is evidence showing that RTFQ PCR has high sensitivity (68%, 95% CI: 62%-74%) and high specificity (100%) in the detection of *Mycobacterium tuberculosis* in CSF [10].

In 2000, Notomi *et al* [13] developed a new LAMP technique. This method has the following characteristics: It does not need special instruments: amplification is performed at a constant temperature, which does not require thermal cycler and detector, and only needs water bath; It has a high specificity: Four primers are designed based on the 6 fragments of the target gene. The reaction will not start if one of primers is unsuitable; It has rapid and efficient amplification: the whole amplification can be done within 60 min; Further, it has a high sensitivity: The low limit of detection is 1-10 copies, which is 2-3 magnitudes higher than that of PCR; Meanwhile, it is relatively simple: All the reagents are added before reaction, and one step nucleic acid amplification is realized; Its identification is convenient: the fluorescence can be observed macroscopically [14].

Currently, *gyrb* and *IS6110* are tested as target genes in the detection of *Mycobacterium tuberculosis* with LAMP [15, 16]. *IS6110* is selected because it is present in multiple copies throughout the genome, and it shows higher sensitivity [17]. In this study, primers were designed based on *IS6110*. Our results showed the sensitivity of LAMP in the diagnosis of TBM was 43.02%, which was markedly higher than those of acid-fast staining (2.91%) and MGIT 960 culture (12.79%) (*χ2* = 75.11, *P* = 0.000; *χ2* = 43.88, *P* = 0.002). Moreover, LAMP had a high specificity (92.86%). These results indicate that LAMP has favorable clinical value in the early diagnosis of TBM. Nagdev *et al* [2] used LAMP in the detection of *Mycobacterium tuberculosis* in CSF, their results showed the sensitivity and specificity of LAMP in the diagnosis of TBM were 88.23% and 80.00%, respectively. That study for the first time employed LAMP in the detection of *Mycobacterium tuberculosis* in CSF, but the sample size was small (*n* = 27). In our study, the sensitivity and specificity of LAMP in the diagnosis of TBM were 43.02% (74/172) and 92.86% (26/28), the sensitivity seemed lower than previously reported [2]. However, our results indicated that LAMP had better performance in the diagnosis of TBM as compared to AFB and MGIT 960 culture. Yet, it had similar clinical accuracy to RTFQ PCR. It should be noted that LAMP is cost-efficient. These are consistent with previous report [2]. More studies are still needed to further investigate the clinical application of LAMP for detection of TBM.

There is evidence showing that LAMP increases the low limit in the detection of *Mycobacterium tuberculosis* by 10 times, as compared to PCR assay [18]. This is possibly due to the fact that six primers are designed to recognize eight distinct regions of the target gene by LAMP [18]. Thus, LAMP is more applicable in samples with small amount of bacteria. In the present study, the sensitivity was comparable between LAMP and RTFQ PCR in the detection of *Mycobacterium tuberculosis* (*χ2* = 2.08, *P* = 0.130). They had favorable consistency (88.5%, Kappa = 0.87). As mentioned above, RTFQ PCR is a modified PCR, and its sensitivity is higher than regular PCR. Although the sensitivities ware similar between RTFQ PCR and LAMP. Notably, detection of *Mycobacterium tuberculosis* with RTFQ PCR would cost at least 5-6 hours, and would require expensive instruments. On the other hand, LAMP only requires a short detection time (1 hour), and it does not require expensive instruments. Thus, LAMP is more applicable.

Our study also revealed that the sensitivity of LAMP was significantly higher than that of MGIT960 culture (*χ2* = 43.88, *P* < 0.002), but was similar to that of RTFQ PCR (*χ2* = 2.08, *P* > 0.130). The consistency between LAMP and RTFQ PCR in the diagnosis of TBM was 88.5% (Kappa = 0.87), which was also higher than that between LAMP and MGIT 960 culture (71.0%, Kappa = 0.73). This might be due to that RTFQ PCR and LAMP are molecular biological methods that detect both living and death bacteria. But the routine bacterial culture is only able to detect live bacteria.

Taken together, LAMP has following advantages in the detection of *Mycobacterium tuberculosis* in CSF: First, it showed a significantly higher positive rate as compared to traditional acid-fast staining; Second, it had a higher positive rate and required shorter time of detection, as compared to MGIT 960 culture; Third, it had similar detection rate and detection time to RTFQ PCR, but it was more convenient to perform, which did not need expensive instruments. Fourth, LAMP results can be observed macroscopically. However, to date, few studies with large sample size have been conducted to investigate the activity of LAMP in the detection of *Mycobacterium tuberculosis* in CSF. More measures should be taken to improve the quality control in the detection of *Mycobacterium tuberculosis* in CSF with LAMP.

## MATERIALS AND METHODS

### Patients

A total of 200 patients with anti-TB therapy or diagnostic anti-TB therapy at Shanghai Pulmonary Hospital from January 2014 and December 2015 were enrolled. They were diagnosed with suspected TBM according to clinical symptoms, signs and imaging findings. CSF was collected. Experiments and the protocols in this study were approved by the Ethics Review Board (ERB) of Shanghai Pulmonary Hospital and Tongji University School of Medicine (Shanghai, China). The written-informed consent was obtained from each enrolled individual. All investigations were conducted according to the principles expressed in the Declaration of Helsinki as well as national/international regulations.

### Mycobacterium tuberculosis detection

#### AFB staining

One mL of CSF per sample was collected for Ziehl-Neelsen acid-fast staining to detect tubercle bacillus as described [19].

#### MGIT960 culture

One mL of CSF per sample was incubated with 4% N-acetyl-L-cysteine (NALC)-NaOH for 20 min, and then the mixture (0.1 mL) was seeded into Acidic Roche medium (BASO Biotech Co., Shanghai, China) in duplicate. This was followed by incubation at 37°C for 4-8 weeks. Results were recorded. BACTEC™ MGIT 960 automatic Mycobacterium culture-drug sensitivity system was purchased from BD (Shanghai, China). CSF was cultured according to the manufacturer's instructions (BACTEC™ MGIT, Shanghai, China).

#### Real-time fluorescent quantitative polymerase chain reaction (RTFQ PCR) analysis

CSF (1.5 mL per sample) was collected for DNA extraction. The DNA was further utilized in the LAMP detection and the RTFQ PCR [11, 12]. Nucleic acid rapid extraction kit (Rongyan Biotech Co, Shanghai, China) was applied to extract DNA from *Mycobacterium tuberculosis*. This kit shall disrupt *M. tuberculosis* and cause the release of nucleic acids. The resultant solution was mixed with porous media. Afterwards, a filter was applied to extract the nucleic acids from the mixture. All the procedures were conducted according to the manufacturer's instructions. The resulting DNA was ethanol precipitated and dissolved in 50 μL of EDTA buffer.

#### LAMP

LAMP reaction was designed using six primers targeting the *Mycobacterium tuberculosis* IS*6110* gene: a forward inner primer (FIP), a reverse inner primer (BIP), two outer primers (F3 and B3), and two loop primers (FLP and BLP). The specific primers were synthesized as follows:

F3: 5′-AGACCTCACCTATGTGTCGA-3′;

B3: 5′-TCGCTGAACCGGATCGA-3′;

FIP:5′-ATGGAGGTGGCCA TCGTGGAAGCCTACGTGGCCTTTGTCAC-3′;

BIP:5′-AAGCCATCTG GACCCGCCAACCCCTATCGTATGGTGGAT-3′;

FLP: 5′-AGGATCCTGCGAGCGTAG-3′;

BLP: 5′-AAGAAGGCGTACTCGACCTG-3′.

#### LAMP amplification

nucleic acids (30 μL per sample) was added to a reaction tube, followed by incubation at 67°C for 40 min. The reaction was thereafter terminated with incubation at 80°C for 5 min by inactivating enzyme.

#### Determination

The reaction tube was shaken several times for mixing the amplification products with the SYBR Green I dye (Shanghai, China) coating on the inner wall of the tube beforehand. The tube was irradiated with ultraviolet, and the presence of green fluorescence is suggestive of positive reaction; No fluorescence indicates negative reaction. The fluorescence was observed macroscopically.

#### RTFQ PCR

*M. tuberculosis*. nucleic acid amplification fluorescence detection kit (Da’an Gene Co., Shanghai, China) was applied for RTFQ PCR in a thermal cycler (DA7600) according to manufacturer's instructions.

### Statistical analysis

The patients’ characteristics and detection results were integrated together. All patients were followed up for at least 6 months. CSF detection results, imaging findings and treatment outcomes after therapy were integrated for the final clinical diagnosis.

Relevant definitions and formulas: (1) sensitivity = true positive (n) / [true positive (n) + false negative (n)] × 100%; (2) specificity = true negative (n) / [true negative (n) + false positive (n)] × 100%; (3) positive predictive value = true positive (n) / [true positive (n) + false positive (n) ] × 100%; (5) negative predictive value = true negative (n) / [true negative (n) + false negative (n)] × 100%; (5) Kappa value was determined as follows.

According to the diagnostic criteria developed by TBM Collaboration group in 2010 [5], CSF samples were collected from patients with definite TBM. SPSS version 17.0 was applied for statistical analysis. The sensitivity, specificity, positive predictive value and negative predictive value of LAMP were calculated. The detection rates were compared with chi square test among different methods. A value of *P* < 0.05 was considered statistically significant. Kappa coefficient was calculated, which was utilized to evaluate the consistencies of LAMP with MIGT960 culture and RTFQ PCR. Kappa coefficient is between −1 and +1, and the higher the Kappa value, the better the consistency is. Kappa coefficient of 0.3-0.7 indicates fine consistency, and that of > 0.7 indicates strong consistency.

## References

[1] Thwaites GE, van Toorn R, Schoeman J (2013). Tuberculous meningitis: more questions, still too few answers. Lancet Neurol.

[2] Nagdev KJ, Kashyap RS, Parida MM, Kapgate RC, Purohit HJ, Taori GM, Daginawala HF (2011). Loop-mediated isothermal amplification for rapid and reliable diagnosis of tuberculous meningitis. J Clin Microbiol.

[3] Joon D, Nimesh M, Saluja D (2015). Loop-mediated isothermal amplification as alternative to PCR for the diagnosis of extra-pulmonary tuberculosis. Int J Tuberc Lung Dis.

[4] Uplekar M, Weil D, Lonnroth K, Jaramillo E, Lienhardt C, Dias HM, Falzon D, Floyd K, Gargioni G, Getahun H, Gilpin C, Glaziou P, Grzemska M (2015). WHO's new end TB strategy. Lancet.

[5] Marais S, Thwaites G, Schoeman JF, Torok ME, Misra UK, Prasad K, Donald PR, Wilkinson RJ, Marais BJ (2010). Tuberculous meningitis: a uniform case definition for use in clinical research. Lancet Infect Dis.

[6] Thwaites G, Chau TT, Mai NT, Drobniewski F, McAdam K, Farrar J (2000). Tuberculous meningitis. J Neurol Neurosurg Psychiatry.

[7] Kumar K, Giribhattanavar P, Chandrashekar N, Patil S (2016). Correlation of clinical, laboratory and drug susceptibility profiles in 176 patients with culture positive TBM in a tertiary neurocare centre. Diagn Microbiol Infect Dis.

[8] Koh WJ, Ko Y, Kim CK, Park KS, Lee NY (2012). Rapid diagnosis of tuberculosis and multidrug resistance using a MGIT 960 system. Ann Lab Med.

[9] Chen P, Shi M, Feng GD, Liu JY, Wang BJ, Shi XD, Ma L, Liu XD, Yang YN, Dai W, Liu TT, He Y, Li JG (2012). A highly efficient Ziehl-Neelsen stain: identifying de novo intracellular Mycobacterium tuberculosis and improving detection of extracellular M. tuberculosis in cerebrospinal fluid. J Clin Microbiol.

[10] Chaidir L, Ganiem AR, Vander Zanden A, Muhsinin S, Kusumaningrum T, Kusumadewi I, van der Ven A, Alisjahbana B, Parwati I, van Crevel R (2012). Comparison of real time IS6110-PCR, microscopy, and culture for diagnosis of tuberculous meningitis in a cohort of adult patients in Indonesia. PLoS One.

[11] Li ZW, Cai S, Liu Y, Yang CL, Tian Y, Chen G, Cao C (2016). Over-expression of Galphai3 in human glioma is required for Akt-mTOR activation and cell growth. Oncotarget.

[12] Gong YQ, Huang W, Li KR, Liu YY, Cao GF, Cao C, Jiang Q (2016). SC79 protects retinal pigment epithelium cells from UV radiation via activating Akt-Nrf2 signaling. Oncotarget.

[13] Notomi T, Okayama H, Masubuchi H, Yonekawa T, Watanabe K, Amino N, Hase T (2000). Loop-mediated isothermal amplification of DNA. Nucleic Acids Res.

[14] Boehme CC, Nabeta P, Henostroza G, Raqib R, Rahim Z, Gerhardt M, Sanga E, Hoelscher M, Notomi T, Hase T, Perkins MD (2007). Operational feasibility of using loop-mediated isothermal amplification for diagnosis of pulmonary tuberculosis in microscopy centers of developing countries. J Clin Microbiol.

[15] Ou X, Li Q, Xia H, Pang Y, Wang S, Zhao B, Song Y, Zhou Y, Zheng Y, Zhang Z, Li J, Dong H, Zhang J (2014). Diagnostic accuracy of the PURE-LAMP test for pulmonary tuberculosis at the county-level laboratory in China. PLoS One.

[16] Piatek AS, Van Cleeff M, Alexander H, Coggin WL, Rehr M, Van Kampen S, Shinnick TM, Mukadi Y (2013). GeneXpert for TB diagnosis: planned and purposeful implementation. Glob Health Sci Pract.

[17] Hwang SH, Im SG, Sung H, Hah SS, Cong VT, Lee DH, Son SJ, Oh HB (2014). Upconversion nanoparticle-based Forster resonance energy transfer for detecting the IS6110 sequence of Mycobacterium tuberculosis complex in sputum. Biosens Bioelectron.

[18] Aryan E, Makvandi M, Farajzadeh A, Huygen K, Bifani P, Mousavi SL, Fateh A, Jelodar A, Gouya MM, Romano M (2010). A novel and more sensitive loop-mediated isothermal amplification assay targeting IS6110 for detection of Mycobacterium tuberculosis complex. Microbiol Res.

[19] Kivihya-Ndugga LE, van Cleeff MR, Githui WA, Nganga LW, Kibuga DK, Odhiambo JA, Klatser PR (2003). A comprehensive comparison of Ziehl-Neelsen and fluorescence microscopy for the diagnosis of tuberculosis in a resource-poor urban setting. Int J Tuberc Lung Dis.

